# Crocin nano-chitosan-coated compound improves anxiety disorders, learning, and spatial memory in Alzheimer’s model induced by beta-amyloid in rats

**DOI:** 10.22038/IJBMS.2024.74823.16247

**Published:** 2024

**Authors:** Gholam Hossein Meftahi, Mohsen khodadadi, Gila Pirzad Jahromi, Masoud Ezami Razliqi, Habib Valipour

**Affiliations:** 1 Neuroscience Research Center, Baqiyatallah University of Medical Sciences, Tehran, Iran; 2 Applied Virology Research Center, Baqiyatallah University of Medical Sciences, Tehran, Iran

**Keywords:** Amyloid-beta, Anxiety, Chitosan, Crocin, Hippocampus, Memory, Nanoparticle

## Abstract

**Objective(s):**

Alzheimer’s disease (AD) is a neurodegenerative disease that results in the gradual breakdown of brain tissue, causing the deterioration of intellectual function and ability. Crocin is a saffron carotenoid compound proven to have excellent neuroprotective and anti-inflammation properties, although it has some limitations such as low stability and bioavailability. Therefore, in the current research, we tried to improve these limitations by using nanotechnology and chitosan as the carrier. Our study examined the therapeutic effects of crocin nano-chitosan-coated compound and compared it with intact crocin in lower dosages than other studies in AD rat models.

**Materials and Methods:**

Encapsulating crocin into chitosan nanoparticles was done through a modified technique to improve its limitations. The AD rat model was induced by bilaterally injecting beta-amyloid (Aβ) peptide into the frontal lobe using a stereotaxic device. To evaluate memory, we conducted the Barnes maze test, and to evaluate anxiety, we used the elevated plus maze test. Also, histological tests were conducted to evaluate neuronal damage in each group.

**Results:**

Crocin nano-chitosan-coated administration significantly improved specific memory indicators compared to the Aβ and other treated groups. A significant decrease in anxiety indicators was detected compared to the Aβ and other treated groups. Finally, the results of hippocampus staining indicated a meaningful difference between the Aβ group and other treated groups, compared to the crocin nano-chitosan-coated group.

**Conclusion:**

Treatment with low dosages of crocin in the nano-coated form exhibited great efficacy in reducing AD’s adverse effects compared to the same dosage of intact crocin.

## Introduction

One of the most prevalent types of dementia and neurologic disorders in old age is Alzheimer’s disease (AD), which begins with accumulation of beta-amyloid peptides in the extracellular spaces between neurons and neurofibrillary tangles (caused by the accumulation of tau peptides) inside the neuron’s bodies (1). AD leads to apoptosis and degeneration in specific brain areas, causing cognitive impairment in behaviors such as anxiety disorders, depression, memory loss, and a decrease in other intellectual abilities (2). The mean survival time expected for AD patients is around 5–8 years, and the prevalence rate is increasing rapidly because of our adverse lifestyle in an aging society (3). It has been evidenced in a variety of studies that degeneration and atrophy of the hippocampus lead to dementia and are mostly concurrent with the early stages of AD, which later leads to memory-forming impairment (4). Aβ aggregation in extracellular and vessel walls could cause toxicity that leads to some area-specific AD lesions (5). For many years, multiple studies have been conducted in search of a medicine that could reduce the progression of AD. Nowadays, AD treatments are based on moderately relieving symptoms by administrating some medicines like N-methyl-D-aspartate receptor antagonists and cholinesterase inhibitors (6-8). Thus, developing a medicine that can slow the progression of AD with minimum side effects is of great importance. In recent years, many studies have reported that crocin, a carotenoid compound of saffron, may improve psychological illnesses such as anxiety and depression and could prevent the destruction of hippocampal cells (9-11). It has been discussed that intraperitoneal administration of crocin has a positive effect on memory and spatial learning in the damaged hippocampus (12, 13). Crocin, the scant carotenoid of saffron, can be dissolved in water. Despite its benefits, this substance has some constraints, including instability at variable pH, oxidative and thermal stresses, rapid absorption, and low bioavailability (14, 15). Therefore, a variety of studies have been conducted in search of a solution to increase the stability range of crocin. These studies demonstrated that using crocin in nanoparticle size and encapsulating it by coating crocin with another compound such as chitosan could remarkably increase crocin’s durability and stability (16-18). Chitosan is a natural and biocompatible polysaccharide. This polymer has been widely examined in brain scaffolds and spinal implants as a carrier of drugs for targeted delivery in neurogenic disorder treatments (19, 20). At the molecular level, chitosan and its biodegradable derivations impose their biological activity on neurons and the blood-brain barriers, which can be useful in the treatment of AD (20, 21). Flexibility in changing the surface of nanoparticles carried by chitosan, the ability to attach a variety of ligand cells, and the establishment of stable nanoparticles under biological conditions make chitosan a suitable compound for delivering AD medications (22). In addition to biodegradability and biocompatibility, chitosan has the flexibility to modify the surface and ease of use with various preparation methods, such as the successful delivery of medicines and nucleic acids by drug nanocarriers to the brain-blood barrier (23). Therefore, in the current research, we aimed to explore the impacts of crocin coated with nano chitosan as a nano complex on the morphology of hippocampal neurons, memory and learning disorders, and anxiety behaviors using lower dosages of crocin compared to other studies. We also aimed to compare it with a similar dosage of intact crocin in the AD rats model induced by beta-amyloid injection.

## Materials and Methods

***Selected animals***

Male Wistar rats (220–250 g) were selected as the subjects for our study, and they were randomly placed into six groups (n=6). We divided each group into two cages containing 3 rats each, with no restriction on food and water, under a 12-hour light and dark cycle. The room temperature was maintained at 25 °C (25±2). Our study was done following the animal welfare rules and in accordance with instructions confirmed by the local and international ethical committees. We took all necessary measures to minimize the number of rats used and to alleviate any potential pain or discomfort. This research was confirmed through the Baqiyatallah University of Medical Science’s ethical committee in Tehran, Iran (Ethics Code: IR.BMSU.REC.1399.599).

***Study’s experimental groups***

In the current experimental study, rats were divided into six groups, each containing six rats. One group received Aβ (1-42) 3 µl at a bilateral concentration of 10 ng/ml, administered into the frontal lobe cortex (referred to Aβ group) (24). Another group received a single dose of Aβ and crocin nano-chitosan-coated powder, dissolved in sterile distilled water at a concentration of 180 mg/kg, and administrated by IP injection for 12 sequential days (referred to as the nanoparticle group). The following are the remaining groups in our experiments: the crocin group (received a single dose of Aβ + crocin, dissolved in sterile distilled water at a concentration of 6 mg/kg, and administrated by IP injection daily for 12 days), the chitosan group (received a single dose of Aβ + chitosan, dissolved in sterile distilled water at a concentration of 160 mg/kg, and administrated by IP injection daily for 12 days), the sham group (underwent the surgical procedure without drug injection), and the control group (did not undergo the surgical procedure and did not receive any drugs).

***Preparation and characterization of chitosan-crocin nanoparticles***

To obtain the crocin nano-chitosan-coated compound, first, a 5 ml solution of 0.2% chitosan in 1% acetic acid is prepared. Additionally, 6 mg of crocin is dissolved in one milliliter of distilled water and then added to the prepared chitosan solution. One milliliter of 0.1% TPP solution is added to the chitosan solution drop by drop while stirring and then the final solution should be stirred for 2 hr. Eventually, the above-mentioned solution was centrifuged for one hour (10000 – 14000 RPM), the supernatant removed, and then the desired sediment was again dispersed in MiliQ water and stored at -20 ℃ for future use or freeze-drying (17). The hydrodynamic particle size was investigated using DLS (SOS I, KONE (South Korea)).

The amount of crocin content in the nanoparticles was evaluated by calculating the difference between the total amount of crocin added to the nanoparticles during the preparation process and the amount of unentrapped crocin in the supernatant. The crocin content was analyzed using a UV–Vis spectrophotometer (Infinite 200 PRO, TECAN, Switzerland) at 440 nm (25). The EE was calculated by the equation below: 

$\mathrm{EE}\left(\%\right)=\frac{\mathrm{TC}-\mathrm{FC}}{\mathrm{TC}}\times 100$

(TC=Total amount of crocin, FC=Free amount of crocin)

Also, the loading capacity was estimated by the following equation:

$\mathrm{LC}(\%)=\frac{\mathrm{TC}-\mathrm{FC}}{\mathrm{wt of nanoparticles retrived}}\times 100$

***Animal surgery***

Animals were anesthetized through a compound drug comprised of xylazine (10 mg/kg, IP) plus ketamine hydrochloride (60 mg/kg, IP) for stereotaxic surgery. After shaving the rats’ heads, they were placed into a stereotaxic instrument, and the surgical procedure began with optimum delicacy. For stereotaxic surgery in the frontal cortex, the coordinates used were according to the Paxinos Atlas and other studies, namely +2.3 mm AP, +2.4 mm DV, and ±3 mm ML relative to the bregma. We administrated a dose of Aβ peptide (1-42) bilaterally (1.5 µl for every side) into the frontal lobe using a Hamilton syringe (24). 

***Barnes maze***

**Figure 1:**
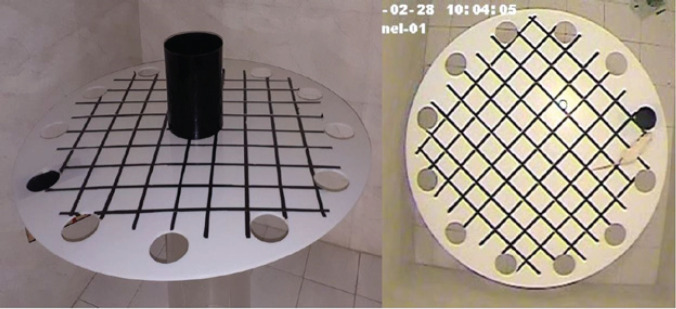
The Barnes Maze test apparatus in rat’s model

After 8 days of stereotaxic surgery, the rats were subjected to behavioral tests. We used the “Barnes maze” test to estimate memory and learning impairment. This maze was made of a 90 cm diameter white Plexiglas plate with 12 holes, each having an 8 cm diameter. The holes were positioned 2 cm away from the edges, with a 5 cm distance between them. Beneath one hole was a dark box (goal chamber) made of black Plexiglas with a diminution of 10 × 10 × 10 cm, providing comfort and reducing stress for the rats (Figure 1). A gram of food was placed in this chamber. Four pieces of painted paper with different colors were placed as surrender signs in the testing room. At a height of 110 cm above the maze plate, two 150 W lamps were placed to serve as evasive stimuli. Before starting the tests, each group of rats underwent an adaptation phase, which involved spending one hour in the testing room. The Barnes test spanned five days in total, involving four days of training and a final day to obtain the conclusive results. During the initial four days of training, the rats learned to use distal visual signs to estimate the spatial location and navigate to the dark chamber. In each trial of the Barnes test, the rats were placed in a dark Plexiglas cylinder at the center of the Barnes maze plate. Once the cylinders was removed, the rats were allowed to freely search and find the escape hole. The parameters we measured for detecting the difference in memory between the groups were the time interval between cylinder removal and reaching the escape chamber, and the number of errors in locating the escape hole. During the training days, we conducted four trials per animal, each lasting one minute. The rats had one minute to locate the escape hole, and if they failed to do so within that time, we would help them until they found it independently. On the fifth day (day 12), the same process was repeated, but only one trial was conducted. After testing each animal, we cleaned the entire maze plate and scape chamber with 70% ethanol to eliminate intra-maze odor (24).

***Elevated plus maze***

The elevated plus maze (EPM) is a behavioral test to evaluate the anti-anxiety effects of medicinal drugs in rodents and specify the brain areas and processes associated with anxiety-related behaviors. The main objective of using this behavioral test in this research is to assess the anti-anxiety effect of crocin nano-chitosan-coated nanoparticles on AD.

A day after ending the Barnes test (day 13), the rats were subjected to the EPM test. EPM consisted of four arms made of black Plexiglas material 50 cm long and 10 cm wide. Two of the four arms were without walls, while the other two arms were closed with a wall height of 30 cm. In the open arms of the EPM, we added an additional fence of 3–5 mm high on each side to increase open-arm exploration. Each arm of the maze was attached to sturdy plastic legs, elevating it 60 cm above the ground. This test consisted of a single trial lasting 5 min for each rat. First, we placed the rats at the intersection of the arms, facing toward the open arm, after which we closed the room door and started filming its movements. The parameters we measured to assess the anti-anxiety effects included open arm time, $\left(\mathrm{\%OAT}=\frac{\mathrm{open arm time}}{\mathrm{open}+\mathrm{close arm time}}\times 100\right)$ open arm entry, $\left(\mathrm{\%OAE}=\frac{\mathrm{open arm entry}}{\mathrm{open}+\mathrm{close arm entry}}\times 100\right)$ grooming, head dipping, and freezing (time latency of staying in one place without movement). After testing each animal, we cleaned the entire maze with 70% ethanol to eliminate any intra-maze odors before the next trial (26).

***Histopathological evaluation***

After conducting the EPM test to evaluate neuronal damages, such as decreases in neuronal dendritic branches and atrophy, the rats were euthanized on day 13. Afterward, we randomly selected 5 rats from each group and performed cresyl-violet (CV) and golgi-cox staining on brain sections obtained from the hippocampus according to previously studied methods (27, 28).

***Injection site confirmation***

**Figure 2:**
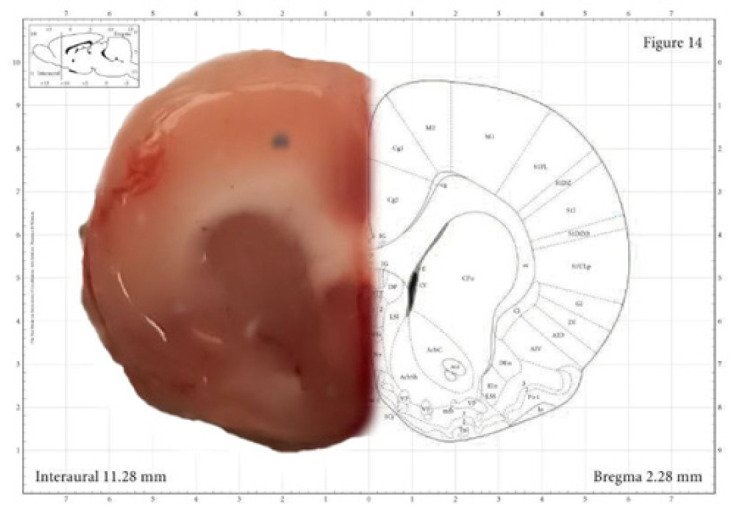
**. **Injection site of Amyloid-beta (Aβ) and the same region shown in the paxinos atlas

The coordinates we used (2.3 mm anterior, 2.4 mm ventral, 3 mm medial, and lateral) have been confirmed by the Paxinos textbook for the frontal lobe cortex region (Figure 2) (29). 

## Results

***Characterization, entrapment efficiency (%) and loading capacity***

According to the preparation method, after centrifuge, 0.9 mg of the designated drug was found to be unbound out of the initial 6 mg. Also, the EE% was calculated using the standard curve of crocin (Table 1), which was plotted based on the UV-VIS absorbance at 440 nm (17). Furthermore, our nanoparticles had a hydrodynamic size of 175±5 nm after preparation.

***Analysis of memory impairment by barnes maze test***

**Figure 3:**
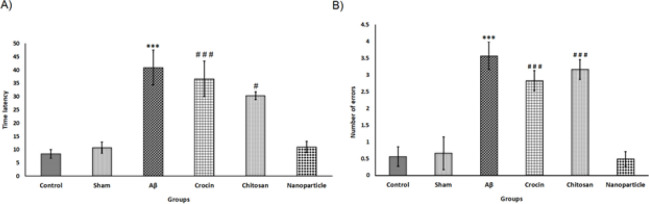
Results of memory and learning evaluated in rats by Barnes maze behavioral test

Eight days after the surgery, at the beginning of the test, all rats showed similar results. However, after four training days on the fifth day, different scales of reduction in time latency to find the goal and errors during the process were observed in all the testing groups. As shown in Figure 3 A, the group that received Aβ into the frontal cortex bilaterally had a significantly longer time latency (40.92±6.56) compared to the control group (8.39±1.64) and the sham group (10.75±2.09). One-way ANOVA revealed significant differences between the groups (F=13.308, df=5), followed by Tukey’s test (*P*<0.001). However, there was no significant difference between the nanoparticle group (11±2.11) and the control and sham groups. On the other hand, the nanoparticle group showed more promising and significant results compared to the crocin (39.66±6.69) (*P*<0.001) and Chitosan (30.33±1.46) groups (*P*<0.05). Regarding the errors, it was observed that the Aβ group had significantly higher errors (3.57±0.42) compared to the control (0.57±0.29) and sham (0.66±0.49) groups. Furthermore, One-way ANOVA and subsequent Tukey’s test showed a significant difference between the Aβ group and the control and sham groups (F=16.69, df=5, *P*<0.001). However, the nanoparticle group results (0.50±0.22) were not significantly different from the control and sham groups. The results revealed that the nanoparticle group had fewer errors compared to the crocin (2.83±0.30) (*P*<0.001) and chitosan (3.16±0.30) (*P*<0.001) groups, and there were significant differences between the nanoparticle group and them.

***Analysis of stress and anxiety levels by elevated plus maze***

**Figure 4:**
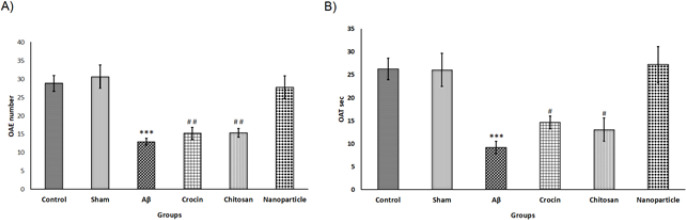
Results of evaluating anxiety parameters in rats using the elevated plus maze

The level of stress and anxiety in testing rats was assessed by the EPM test. The parameters indicating anxiety, such as OAT and OAE, as well as other behavioral actions including head dipping, freezing, and grooming, were calculated in this study. As shown in Figure 4 B, the OAT in the Aβ group (9.18±1.33) was significantly less than those in the control (26.28±2.34) and sham (26.09±3.64) groups. This difference was confirmed by a one-way ANOVA test (F=8.87, df=5), followed by Tukey’s test, which indicated that the anxiety level of rats that received Aβ bilaterally was significantly larger than in the control (*P*<0.001) and sham (*P*<0.001) groups. However, the OAT data from the nanoparticle group (27.27±3.93) showed an improvement in anxiety compared to the Aβ group (*P*<0.001), and there was no significant difference in the OAT of the nanoparticle group compared to the control and sham groups. As shown in Figure 4, the nanoparticle group exhibited better results compared to the crocin (*P*<0.05) and chitosan (*P*<0.05) groups.

As shown in Figure 4 A, the OAE of the Aβ (12.95 ± 0.96) group was significantly (F=13.58, df=5) different from those of the control (28.86±2.12) (*P*<0.001) and sham (30.66±3.16) (*P*<0.001) groups. However, the OAE for the nanoparticle group (27.78±3.06) was not significantly different from those for the control and sham groups, but it was significantly different from those of the Aβ group (*P*<0.001) and other treatment groups (crocin (*P*<0.01) and chitosan (*P*<0.01)).

**Figure 5:**
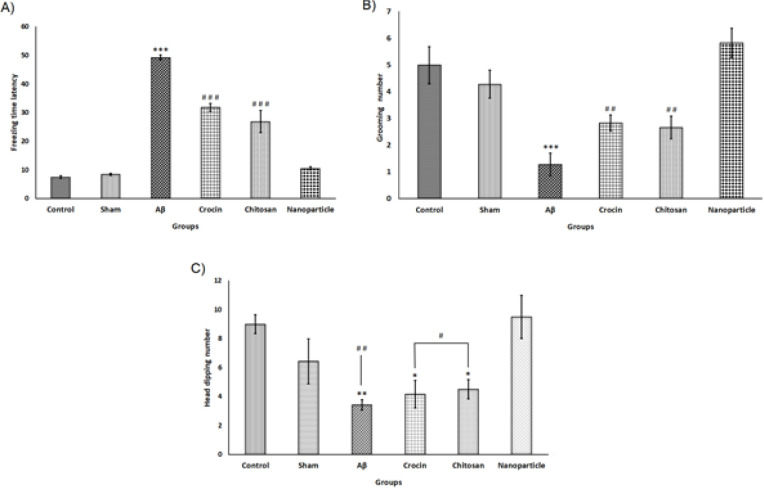
Results of evaluating anxiety parameters in rats using elevated plus maze

As shown in Figure 5 A, the freezing latency for the Aβ group (49.14±0.91) was significantly (F=110.21, df=5) greater than those of the control (7.42±0.48) (*P*<0.001) and sham (8.42±0.42) (*P*<0.001) groups. However, the freezing time in the EPM test for the nanoparticle group (10.50±0.61) was not significantly different from the control and sham groups, but it was significantly different from the Aβ group (*P*<0.001) and other treatment groups.

For the grooming behavior, a one-way ANOVA revealed a significant difference between groups (F=11.00, df=5). The results of Tukey’s test showed that the Aβ group’s (1.28±0.42) number of grooming was significantly fewer than the control (5.00±0.69) (*P*<0.001) and sham (4.28±0.52) (*P*<0.001) groups (Figure 5 (B)). However, the nanoparticle group’s (5.83 ± .54) result did not show a significant difference from the control and sham groups. Furthermore, the nanoparticle group’s result was more promising than those of the crocin (*P*<0.01) and chitosan (*P*<0.01) groups. 

After analyzing the data for head dipping behavior by one-way ANOVA (F=6.17, df= 5) and conducting Tukey’s test, significant differences between groups were observed (Figure 5 (C)). As shown in Figure 5, the number of head dippings in the Aβ group (3.42±0.36) were fewer than those of the control (9.00±0.65) and sham (6.42±1.54) groups, and there was a significant difference between the Aβ group and the control (*P*<0.01) group. However, the nanoparticle group’s results (9.5±1.5) showed no significant difference compared to the control and sham groups, but it was significantly different from the Aβ group (*P*<0.01) and the other two treatment groups (crocin (2.83±0.30) (*P*<0.05) and chitosan (2.66±0.42) (*P*<0.05) groups). It means that our nanoparticle compound had better effects on improving anxiety and its behavioral aspects. 

***Analysis of neuronal damages in the hippocampus by golgi-cox staining***

**Figure 6:**
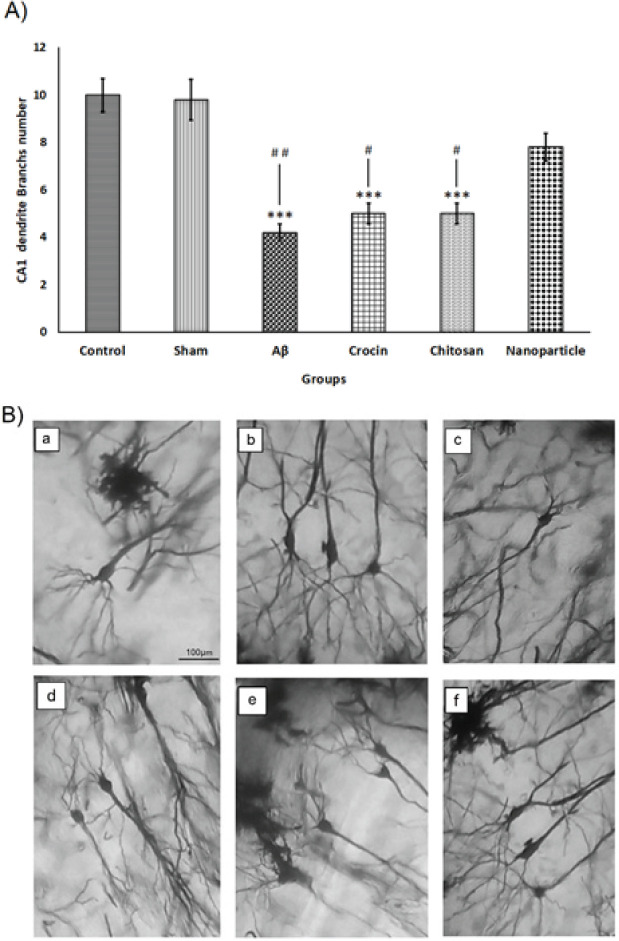
Results of neuronal damages in the CA1 area of the hippocampus in rats that was analyzed by Golgi-Cox staining placed at 40× magnification

**Figure 7:**
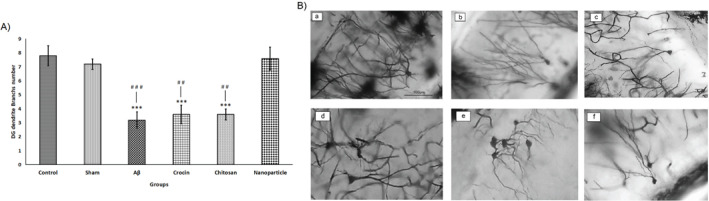
Results of neuronal damages in the DG area of the hippocampus in rats that was analyzed by Golgi-Cox staining placed at 40× magnification

After examining the Golgi-cox staining results from the hippocampus sections, it was shown that in the CA1 and DG regions of the hippocampus in the Aβ group, the number of branches along the cell body branches (dendrites) was significantly decreased (CA1=4.2±0.37; DG=3.2±0.58) compared to the control (CA1=10±0.70; DG=7.8±0.73) and sham (CA1=9.8±0.86; DG=7.2±0.32) groups (CA1: F=18.85, df=5; DG: F=13.09, df=5, *P*<0.001). However, with the intraperitoneal administration of the crocin nano-chitosan-coated compound (180 mg/kg), no significant decrease in dendrite branches was seen, and the results of the nanoparticle group (CA1= 7.8 ± .58) (DG= 7.6 ± .81) did not show a meaningful difference compared to the control and sham groups. As shown in Figures 6 A and 7 A, the nanoparticle group showed more promising results than the crocin and chitosan groups (CA1: *P*<0.05; DG: *P*<0.01), which can also be seen in Figures 6 B and 7 B by comparing the branches along the dendrites in each group.

***Analysis of cresyl violet staining***

**Figure 8:**
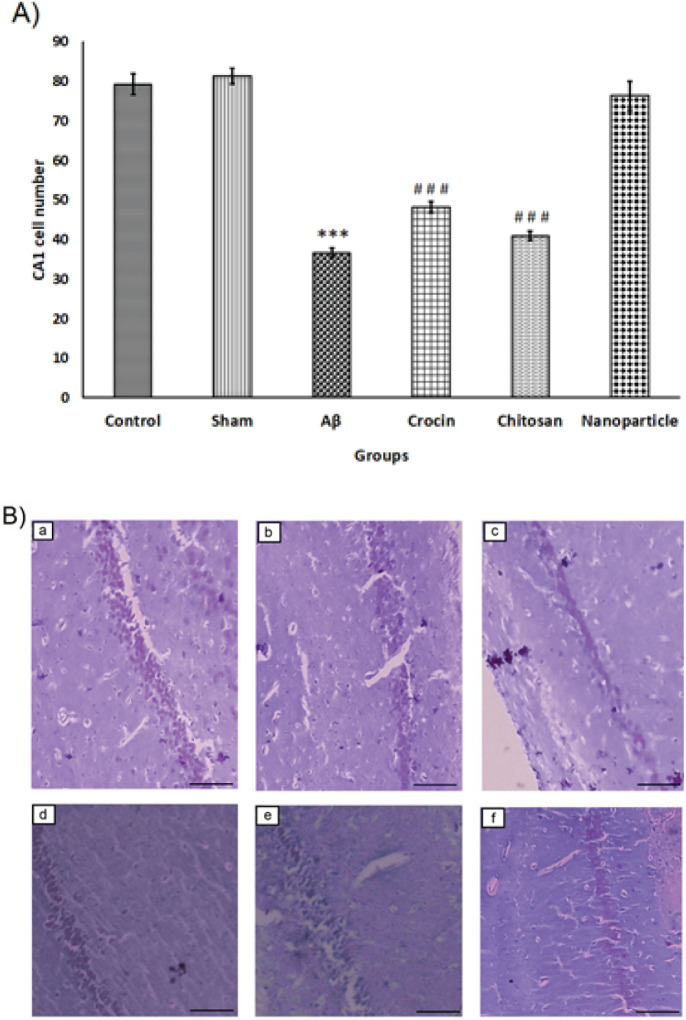
The mean ± SEM number of CA1 the Cresyl violet positive (CV+) cells placed at 20× magnification have been reported in all six experimental groups in rats

Regarding the cresyl violet (CV) staining results, as shown in Figure 8, the number of CV+ cells in the CA1 region of the hippocampus was significantly decreased in the Aβ group (36.60±1.20) compared to the control (79.20±2.67) and sham (81.40±2.01) groups. The significant difference between the groups was confirmed with one-way ANOVA (F=86.95, df=5), followed by Tukey’s test (*P*<0.001). There was no significant difference in the number of CV+ cells in the CA1 region in the nanoparticle group (76.40 ± 3.66) compared to the control and sham groups. The staining results showed a meaningful improvement compared to the crocin (48.20±1.39) and chitosan (41±1.22) groups (*P*<0.001). 

**Figure 9:**
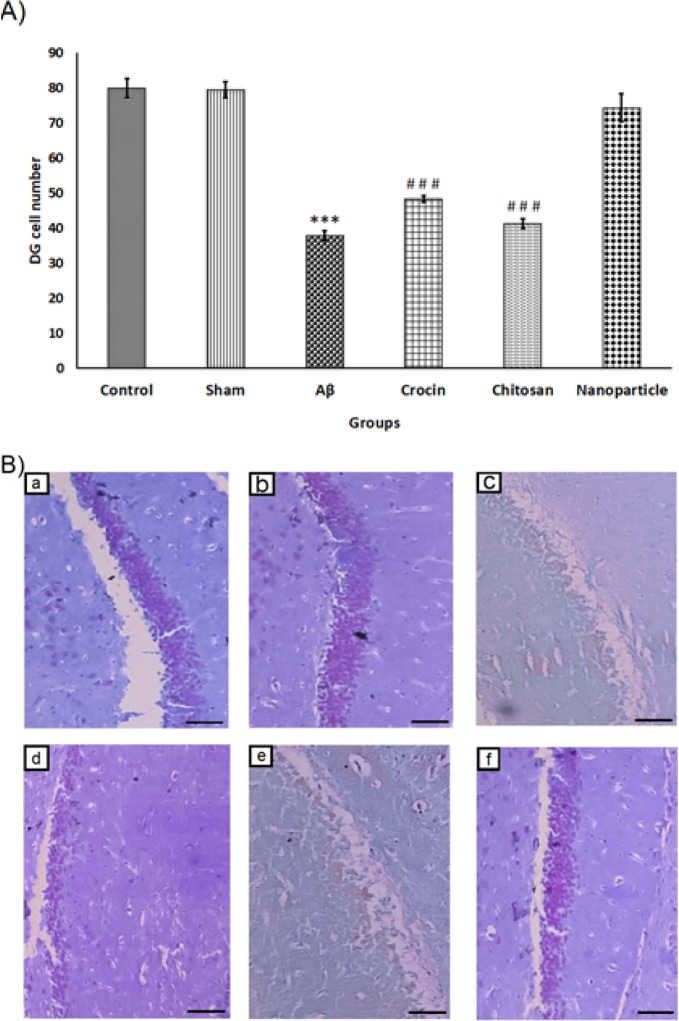
The mean ± SEM number of DG CV+ cells placed at 20× magnification have been reported in all six experimental groups in rats

As shown in Figure 9 A, the number of CV+ cells in the DG area of the hippocampus was significantly decreased in the Aβ group (38±1.41) compared to the control (80±2.73) and sham (79.6±2.33) groups (F=70.59, df=5, *P*<0.001), which can be also seen in Figure 9 B. No significant difference was found between the nanoparticle group (74.4 ± 3.94) and the control and sham groups. However, there was a significant difference between the nanoparticle group and the crocin (48.4±0.92) and chitosan (41.4±1.36) groups (*P*<0.001). 

**Table 1:** Amount of calculated crocin nanoparticles, entrapment efficiency (EE %), and loading capacity (LC %)

Nanoparticle Size (nm)	Entrapment Efficiency (%)	Loading Capacity (%)
**175 ± 5 nm**	85 %	25%

Mean ± SEM of time latency and errors in finding the goal chamber on the fifth day (testing day) have been reported in all six experimental groups. *** = (*P*<0.001) indicates a significant difference between the experimental groups and the control group. # = (*P*<0.05), and # # # = (*P*<0.001) indicates a significant difference between the experimental groups and the nanoparticle group

Mean ± SEM of A) Open arm entry (OAE) and B) Open arm time (OAT) have been reported in all six experimental groups. *** = (*P*<0.001) stands for significant differences between the experimental groups and the control group. # # = (*P*<0.01) and # = (*P*<0.05) show a significant difference between the experimental groups and the nanoparticle group.

Mean ± SEM of A) grooming, B) head dipping, and C) freezing has been reported in all six experimental groups. *** = (*P*<0.001), ** = (*P*<0.01), and * = (*P*<0.05) stands for significant differences between the experimental groups and the control group. # # # = (*P*<0.001), # # = (*P*<0.01), and # = (*P*<0.05) shows a significant difference between the experimental groups and the nanoparticle group.

The mean ± SEM number of CA1 neuronal dendrites and dendrite branches has been reported in all six experimental groups: a) control group, b) sham group, c) Aβ group, d) crocin group, e) chitosan group, and f) nanoparticle group. *** = (*P*<0.001) indicates a significant difference between the experimental groups and the control group, # # = (*P*<0.01) and # = (*P*<0.05) stand for a significant difference between the experimental groups and the nanoparticle group.

The mean ± SEM number of DG neuronal dendrites and dendrite branches has been reported in all six experimental groups: a) control group, b) sham group, c) Aβ group, d) crocin group, e) chitosan group, and f) nanoparticle group. *** = (*P*<0.001) indicates a significant difference between the experimental groups and the control group, # # # = (*P*<0.001) and # # = (*P*<0.01) stands for a significant difference between the experimental groups and the nanoparticle group

(a) Control group, (b) Sham group, (c) Aβ group, (d) Crocin group, (e) Chitosan group, (f) Nanoparticle group. *** = (*P*<0.001) shows a significant difference between the experimental groups and the control group and # # # = (*P*<0.001) indicates a significant difference between the experimental groups and the nanoparticle group.

(a) Control group, (b) Sham group, (c) Aβ group, (d) Crocin group, (e) Chitosan group, and (f) Nanoparticle group. *** = (*P*<0.001) shows a significant difference between the experimental groups and the control group and # # # = (*P*<0.001) indicates a significant difference between the experimental groups and the nanoparticle group.

## Discussion

Numerous studies have been conducted on crocin, documenting its neuroprotective and anti-inflammation effects on different neurological illnesses, including AD (30-32), Parkinson’s disease (33, 34), memory loss, damage and deterioration (35, 36), as well as anxiety and depression (10, 37, 38). Several studies have demonstrated that after 12 days of administrating Aβ into the frontal cortex, neuronal damage and loss can be seen in CA1 and other regions of the hippocampus, which are distant from the injection site (24, 39).

On the other hand, recent studies have demonstrated that these effective properties of crocin come with some limitations, such as low stability and bioavailability. Therefore, finding a way to improve crocin’s effectiveness could open up a great opportunity to use this compound more effectively in the treatment of neurodegenerative disorders such as AD. A very novel approach could be the preparation of crocin with nanoparticles and using a carrier (17). As an example, one of the carriers that has been mostly used in variant studies is chitosan, which has perfect properties like biocompatibility, non-toxicity, a wide range of applications, good stability, and efficient carrier for loading and releasing the drug in designed sites to overcome crocin’s limitations (17, 18, 40, 41). Therefore, we used a modified method to coat crocin with chitosan at the nanoparticle level to improve crocin’s loading in target sites, increase its stability and bioavailability, and enhance its penetration through the blood-brain barrier.

According to other studies and to our knowledge, no research has been done on the crocin nano-chitosan-coated compound in an AD model in rats using a bilateral injection of Aβ in the frontal lobe to induce AD (24, 42, 43) and measure its neuronal protection, as well as comparing it to crocin. 

In comparison to previous studies where significant results were obtained using intact crocin (30 mg/kg) for AD (24, 43), we did not achieve significant results when using intact crocin with a lower dosage (6 mg/kg) in rats with AD. However, when using the same dosage of crocin (6 mg/kg) coated with nano-chitosan as a nano-complex compound, we obtained significant outcomes in behavioral and histological tests. 

The results of the Barnes behavioral test, which was performed on the eighth day after surgery and the injection of Aβ peptide in the frontal region, showed impairments in memory quality and spatial learning. Similar findings of memory impairment due to Aβ have been confirmed in other studies through various behavioral tests (44). Furthermore, as demonstrated in both other studies and our own research, the results of the EPM behavioral test revealed that bilateral injection of Aβ in the frontal lobe after 12 days, can induce anxiety and stress states, characteristic of AD, (43). In addition, we intended to measure histological changes in the structure and quantity of neurons after Aβ injection, as well as its toxic effects on specific areas in the hippocampus. Moreover, we aimed to explore the therapeutic properties of crocin nano-chitosan-coated compounds in the CA1 and DG areas (43, 45).

Intraperitoneal injection of crocin nano-chitosan-coated compound was administered to observe its therapeutic properties on AD, which was caused by bilateral injection of Aβ into the frontal lobe of the brain. This treatment started 12 hr after Aβ injection and continued daily for 12 consecutive days at a specific dose (180 mg/kg). The treatment resulted in a considerable enhancement in memory, learning, and spatial perception as observed in the Barnes Maze Test. Additionally, there was a significant reduction in anxiety levels as measured in the EPM Test, as well as an improvement in the toxic effects of Aβ in brain tissue compared to the administration of intact crocin with a specific dosage of 6 mg/kg and chitosan with a specific dosage of 160 mg/kg.

The results of the nanoparticle treatment group showed a significant reduction in freezing behavior. Also, this treatment was observed to have positive effects on general anxiety behaviors, such as an increase in head dipping. On the other hand, the groups treated with crocin and chitosan showed improvements in general anxiety behaviors, a decrease in freezing, and an increase in head dipping behaviors compared to the Aβ group, although no significant differences were observed. Furthermore, the nanoparticle group showed a significant increase in the time spent on the OAT and the number of entries into the OAE compared to the Aβ group. These superior results of the crocin nano-chitosan-coated compound are likely due to its greater ability to penetrate the Blood-Brain barrier (BBB) and its higher stability in the nano-chitosan-coated form, as compared to intact crocin. Moreover, it has a stronger effect in increasing anti-oxidants and reducing oxidative mechanisms (46). The greater effect of the study compound is also attributed to its positive effects on the dopaminergic system and norepinephrine inhibitors (46).

In terms of spatial memory and learning, the results of the Barnes maze behavioral test revealed that in the nanoparticle group, the time taken by rats to reach the destination hole and the number of errors in finding it were significantly reduced compared to the Aβ group. These results did not differ significantly from the sham and control groups. However, the mentioned parameters were also reduced in the crocin and chitosan treatment groups, but they did not significantly differ from the Aβ group. These results are likely attributed to the compound under study having better penetration into the BBB and being more stable than intact crocin, resulting in improved effects on increasing the amount of acetylcholine and decreasing acetylcholinesterase in the hippocampus. However, the precise mechanism of this action requires further investigation (47).

According to the histological results, examination of the Golgi-cox staining revealed a decrease in the dendrites and their branches in the CA1 and DG areas of the hippocampus in the Aβ group. However, these decreases were significantly reduced in the nanoparticle group compared to the Aβ group, yielding better results compared to the other therapeutic groups. Additionally, examination of the crystal violet staining results revealed a higher number of CV + cells in the CA1 and DG areas of the nanoparticle group compared to the crocin and chitosan groups. Although a significant difference was observed between the nanoparticle group and the Aβ group, no significant differences were seen between the crocin and chitosan groups compared to the Aβ group. As shown in other studies, cell death and reduction of neuronal dendrites in the hippocampus are believed to be factors contributing to anxiety-like behaviors, which are in line with our findings (48). However, further investigation is needed to determine the mechanism behind crocin’s effect on cell morphological changes and cell death.

## Conclusion

Taken together, the outcomes of our study show that using the crocin nano-chitosan-coated compound after Amyloid-Beta injection can reduce memory impairment, anxiety levels, and neuronal loss in the hippocampus region of the brain. Additionally, superior results were obtained in the nanoparticle group compared to the crocin and chitosan groups. Moreover, our results suggest that the limitations of crocin in the form of nano-complex were reduced and its effectiveness was enhanced. This study was designed and implemented to investigate a more practical way to improve crocin’s limitations in order to find an efficient new practical treatment for reducing the symptoms of AD with fewer side effects to contribute to advancing knowledge and finding the mechanisms involved in the functioning of this disease.

## Authors’ Contributions

GH M and G PJ conceived and designed the study and supervised; M K did the experiments and the statistical analysis and wrote the manuscript. M K and H V participated in collecting data. M ER helped with preparation of the nanoparticle.

## Data Availability

Data will be made available upon request.

## Conflicts of Interest

The authors declare that they have no conflicts of interest.
